# Development and validation of a robust automated analysis of plasma phospholipid fatty acids for metabolic phenotyping of large epidemiological studies

**DOI:** 10.1186/gm443

**Published:** 2013-04-25

**Authors:** Laura Yun Wang, Keith Summerhill, Carmen Rodriguez-Canas, Ian Mather, Pinal Patel, Michael Eiden, Stephen Young, Nita G Forouhi, Albert Koulman

**Affiliations:** 1 Medical Research Council Human Nutrition Research, Elsie Widdowson Laboratory, 120 Fulbourn Road, Cambridge, CB1 9NL, UK; 2 MRC Epidemiology Unit, Institute of Metabolic Science, Box 285, Addenbrooke's Hospital, Hills Road, Cambridge, CB2 0QQ UK

## Abstract

A fully automated, high-throughput method was developed to profile the fatty acids of phospholipids from human plasma samples for application to a large epidemiological sample set (*n *> 25,000). We report here on the data obtained for the quality-control materials used with the first 860 batches, and the validation process used. The method consists of two robotic systems combined with gas chromatography, performing lipid extraction, phospholipid isolation, hydrolysis and derivatization to fatty-acid methyl esters, and on-line analysis. This is the first report showing that fatty-acid profiling is an achievable strategy for metabolic phenotyping in very large epidemiological and genetic studies.

## Background

The association between the fatty-acid composition of the diet and the development of various diseases has been the subject of many epidemiological studies. It is also recognized that gene-diet interactions may play an important role in the development of chronic disease. The ability to study these associations with sufficient certainty relies on very large sample sets. Most past studies used fatty-acid composition information derived from self-reported dietary questionnaires, which can be prone to substantial measurement error and may contribute to inconclusive results across different studies, for example the association between specific fatty acids and type 2 diabetes [1-4]. This has led to the search for nutritional biomarkers for fatty acids that could be used to validate the data obtained by dietary questionnaires, or to be used to investigate gene-diet interactions directly. Fatty-acid biomarkers are regarded to provide an objective measurement of some types of dietary fat intake [5], and epidemiological studies can use fatty acids measured in several blood lipid pools as nutritional biomarkers to investigate the association with disease conditions, such as type 2 diabetes [6,7]. However, the lack of published methods to profile fatty acids in very large sample sets has hampered efforts to combine metabolic information with genetic data to study gene-diet interaction in disease development. Although several studies have combined genetic data with metabolic profiling, most of these studies did not focus on gene-diet interactions in relation to disease development, used only data from healthy individuals [8,9], did not have sufficient power to allow investigation of the association with diet [10], or did not find an effect of diet on the association between fatty acid levels and genes[11].

The application of nutritional biomarkers in large-scale epidemiological studies can be possible only if the analysis of the biomarkers is sufficiently fast, relatively cheap, robust and precise. We decided to to develop an automated method specifically for fatty-acid profiling of the plasma-phospholipid fraction, which would provide the molar percentage of each of the fatty acids in line with previous reports [5-7,11]. This automated method comprised the following steps: 1) extraction of total lipids from plasma samples; 2) isolation of the phospholipid fraction from the total lipids by solid-phase extraction (SPE); 3) a combined hydrolysis and derivatization step to convert the phospholipids into free fatty acids and then methylate these to form the more volatile fatty-acid methyl esters (FAME); 4) FAME extraction and 5) the final gas chromatographic separation combined with flame ionization detection. The chromatographic separation was developed to resolve different geometric isomers of particular fatty acids such as *cis *and *trans *oleic acid (C18:1n9) in an acceptable chromatographic run time. However, these complex sample -preparation procedures for fatty-acid analysis are time-consuming and allow the possibility of errors when performed manually.

Several groups have reported the development of automated or high-throughput fatty-acid profiling of human plasma samples [12,13]. However, none of these methods automated both the phospholipid extraction and FAME derivatization and applied this to very large sample sets [14]. Since fatty-acid profiling by gas chromatography (GC) using FAME derivatization first became established, improvement in sample throughput has been sought, and this is still an active area of research [15,16]. This study is the first report of an automated method for fatty-acid analysis of the phospholipid fraction of human plasma, which includes both phospholipid extraction and FAME derivatization, and has been successfully applied to date to more than 28,000 samples of the InterAct project (detailed information about the subjects can be found in the cohort description [17]). The InterAct project aims to measure the plasma-phospholipid fatty-acid profile in 12,403 verified diabetes cases and 16,154 subcohort participants within the European Prospective Investigation into Cancer study, in eight European countries [17]. Achieving this aim is not practical with standard manual sample preparation [6], therefore, our aim was to develop a completely automated, high-throughput method that would give results comparable with those of previous studies that used manual methods. This automated method should enable conduct of large epidemiological studies such as the InterAct project, covering 28,000 samples [17].

In this report, we focus on aspects of method development, validation, and quality control (QC). The method has been validated on different batches of QC samples. The actual analysis is carried out on three parallel systems, each consisting of two robots and a GC system, which work in parallel using exactly the same equipment and methods. This system is unique in its ability to run very large sample sets, and in this paper, we report the method validation and the results obtained for the QC material used for the first 860 batches analyzed by the method, which demonstrates the ability of the apparatus to maintain the same quality over many samples using the three independent systems. This report is a quintessential first step in the process of applying metabolic phenotyping to study gene-diet interaction in the development of type 2 diabetes [17].

## Results and Discussion

### Comparison of conventional with automated sample preparation

**Figure 1:**
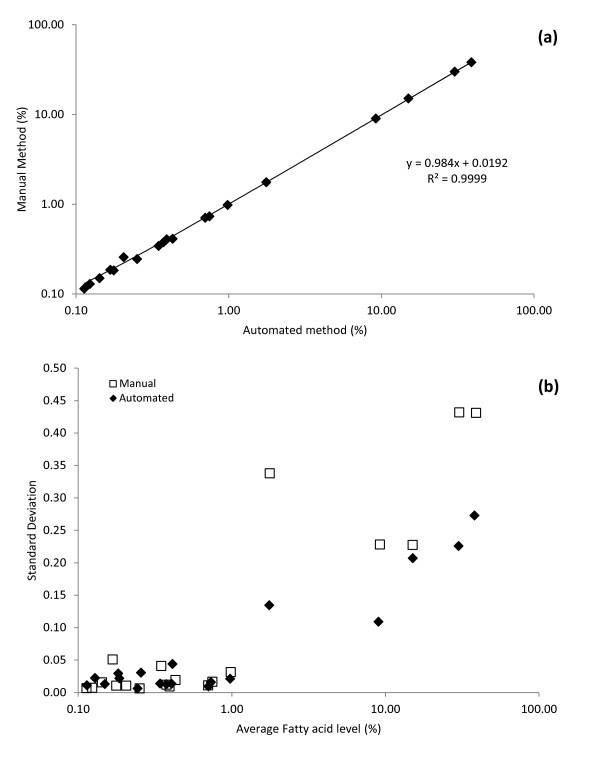
**Accuracy and precision of the automated sample-preparation method compared with the conventional manual method**. **(a) **Correlation of mean values (%) of 19 fatty acids (Table S1) measured by conventional (*n *= 26) and automated (*n *= 26) methods. **(b) **Scatterplot of the fatty-acid average against the standard deviation for both methods, showing the differences in precision (*n *= 26 both groups; based on Van Batenburg *et al. *[23]).

Comparison experiments were conducted using 52 identical QC2 (horse plasma) samples divided randomly into two groups. The aim of this experiment was not to revalidate the approach of using SPE extraction in combination FAME analysis by GC to determine the fatty-acid composition of the phospholipid fraction; this approach, which can have qualitative and quantitative limitations, has already been widely used in nutritional and epidemiological studies [5]. Our aim was to determine if the automated method could deliver results that are comparable with manual methods. Extraction of total lipids and isolation of the phospholipid fraction were performed by both conventional (*n *= 26) and automated (*n *= 26) methods. The mean, standard deviation (SD) and percentage coefficient of variation (%CV) of 19 major phospholipid fatty acids (for peaks over 0.1%, horse-plasma phospholipids are less diverse than human plasma phospholipids) in the QC2 samples were calculated from the two methods (Figure 1; see Additional file 1, Table S1). The mean values for the 19 fatty acids obtained with the automated method showed high correlation (r = 0.9999) with those performed with the manual method, indicating a significant linear relationship (*P *< 0.001) (Figure 1A). However, the precision of the automated method was better than that of the manual method with SDs of less than or equal to those of the manual method (Figure 1B). The Bland-Altman plot showed good agreement between the two methods, with an average difference of 0.03%. shows The automated method had smaller %CV values than those of the conventional manual method for most of the fatty acids, indicating that the analytical precision was improved by the use of the newly developed automated method (see Additional file 1, Table S1).

### Method validation

The flame ionization detector (FID) is a sensitive detector for FAMEs, and shows linearity over a wide range of concentrations [18]. In this particular experiment, the dynamic range stretched from 0.15% to 40%. The response for the 37 FAME components showed good linearity (all *R*^2 ^values between 0.991 and 1.00) (see Additional file 1, Table S2). The long-term inter-instrument precisions (%CV) of the 37 FAME standards have been validated by daily monitoring of two different levels of calibration standards analyzed on three different instruments between 56 runs, shown in Supporting Information (see Additional file 1, Table S2). The detection limits from 0.3 to 1.8 ng per injection for the 37 FAMEs were calculated (see Additional file 1, Table S2), based on a signal-to-noise ratio of 3:1. The limit of quantification (LOQ) for plasma samples with a volume of 125 μl (2 μl GC injection) or 250 μl (1 μl GC injection) were also calculated (see Additional file 1, Table S2). The 250 μl volume was selected for routine analysis of plasma samples and the 125 μl volume was used when repeats were required.

The analytical accuracy and intra-assay %CV within batches were examined by analyzing the six plasma replicates (250 μl) spiked with known concentrations of C15:0 (330 μmol/l) and *cis *C18:2n6 (570 μmol/l) phosphatidylcholines. The recoveries ranged from 77.1% to 89.3% with a %CV of 5.5 for C15:0, and from 83.3% to 100.8% with a %CV of 10.0 for *cis *C18:2n6 (for detailed results, see Additional file 1, Table S3).

Not all of the 37 fatty acids were present in the QC material at sufficient levels to be reproducibly measured (Table 2). All fatty acids present at levels of less than 0.15% showed insufficient reproducibility (CV > 20%). The precision for these low-level fatty acids was not an issue, as the standards mixture held sufficient amounts of these fatty acids for reproducibility to be satisfactory (CV < 5%). As a general rule, all measurements with a level of less than 0.15% were assessed as being below LOQ. Although a splitless injection could have increased the LOQ, it would also have decreased the throughput because it would have required a different temperature program to ensure adequate resolution of the solvent peak from the shorter-chain FAMES.

### Internal quality control in routine analysis

**Figure 2:**
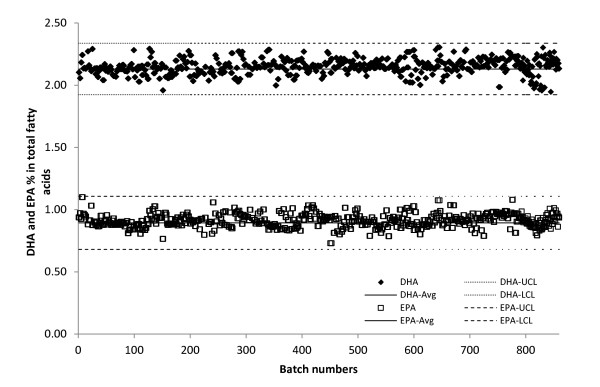
**The Shewhart quality control (QC) chart of 20-month individual measurements for phospholipid docosahexaenoic acid (DHA) (C(22,6n3)) and eicosapentaenoic acid (EPA) ((C(20,5n3)) in the QC material (QC1)**.

Analysis of this very large sample set took place over several months. It required a rigorous internal QC (IQC) system to ensure that the measured fatty-acid results were reliable and that data across the whole study were comparable. IQC was conducted by inserting QC1 and QC2 materials into each batch of analysis. After preliminary replicated QC1 and QC2 results in 60 successive batches were collected, the estimated mean and SD between batches were used to build the QC charts for each of the fatty-acid components. The QC1 and QC2 materials were successively analyzed daily over a 20-month period. The QC1 and QC2 measurements within these 860 batches were obtained from three parallel automated systems. Shewhart control charts were used [19], which include an average line (Avg), a lower control limit (LCL) and an upper control limit (UCL) to determine if data obtained within a batch were comparable with previously obtained data. An example of the Shewhart control chart with individual measurements of DHA (C22:6n3) and EPA (20:5n3) in human plasma (QC1) is shown in Figure 2. There were no batches for which the obtained data were outside the QC limits for either the DHA or EPA results over 860 batches. The long-term reproducibility and stability of the control materials were verified over a period of 20 months and presented as %CV. For instance, over 860 analyses of the human plasma QC1 sample, the mean DHA percentage was 2.15% with CV = 2.8%, and the mean EPA percentage was 0.47% with a CV = 6.8%. These DHA and EPA results were divided into 10 monthly subgroups. The monthly means were compared bysingle-factor ANOVA (Table 1). There were no statistically significant differences (all *P *> 0.05) between the monthly means, and no evidence for any trend towards difference over a period of 10 months for both DHA and EPA measured in human plasma QC1.

**Table 1:** Comparison of the monthly mean percentages of the docosahexanomic acid (DHA) and eicosapentaenoic acid (EPA) profiles.

Months	DHA			EPA		
	Mean	SD	**%CV**^**a**^	Mean	SD	%CV
1 (*n *= 36)^b^	2.14	0.06	2.60	0.46	0.02	4.22
2 (*n *= 36)	2.14	0.06	2.66	0.46	0.01	2.22
3 (*n *= 38)	2.13	0.06	3.02	0.47	0.03	7.28
4 (*n *= 38)	2.15	0.08	3.58	0.47	0.04	9.16
5 (*n *= 37)	2.16	0.06	2.66	0.47	0.03	7.13
6 (*n *= 38)	2.15	0.05	2.47	0.48	0.04	8.46
7 (*n *= 38)	2.16	0.04	1.78	0.47	0.03	6.54
8 (*n *= 38)	2.15	0.07	3.23	0.47	0.02	5.30
9 (*n *= 38)	2.15	0.06	2.71	0.46	0.03	6.90
10 (*n *= 38)	2.17	0.05	2.55	0.46	0.03	6.31

Comparisons of means^c^	*P *= 0.062			*P *= 0.064		

^a^Coefficient of variation.

^b^n is the number of batches in the month.

^c^Single-factoR ANOVA.

**Table 2:** Measured levels of all fatty acids across 860 batches analyzed on three independent systems.

	Fatty acid	**Mix Std1**^**a**^			**QC1**^**b**^			**QC2**^**c**^		
		**Mean**^**d**^	**SD**^**e**^	**CV**^**f**^	**Mean**^**d**^	**SD**^**e**^	**CV**^**f**^	**Mean**^**d**^	**SD**^**e**^	**CV**^**f**^
**C8:0**	Caprylic	2.61	0.14	5.36	ND^g^	-	-	ND^g^	-	-
**C10:0**	Capric	3.72	0.15	4.12	0.01	BLOQ^h^	-	0.01	BLOQ^h^	-
**C11:0**	Undecanoic	2.01	0.07	3.50	ND	-	-	0.01	BLOQ	-
**C12:0**	Lauric	4.21	0.13	3.13	0.02	BLOQ	-	0.01	BLOQ	-
**C13:0**	Tridecanoic	2.19	0.06	2.82	0.02	BLOQ	-	0.11	BLOQ	-
**C14:0**	Myristic	4.41	0.10	2.35	0.25	0.04	15.90	0.18	0.04	21.60
**C14:1**	Myristoleic	2.16	0.06	2.69	ND	-	-	ND	-	-
**C15:0**	Pentadecanoic	2.23	0.05	2.06	0.16	0.03	16.07	0.13	BLOQ	-
**C15:1**	Pentadecenoic	2.16	0.05	2.35	ND	-	-	0.01	BLOQ	-
**C16:0**	Palmitic	6.65	0.19	2.89	30.12	0.43	1.43	15.02	0.35	2.30
**C16:1**	Palmitoleic	2.04	0.04	2.13	0.36	0.03	8.53	0.33	0.03	8.55
**C17:0**	Heptadecanoic	2.12	0.06	2.90	0.41	0.03	7.23	0.68	0.04	5.46
**C17:1**	Heptadecenoic	2.22	0.04	2.02	0.07	BLOQ	-	0.11	BLOQ	-
**C18:0**	Stearic	4.40	0.13	3.01	16.18	0.31	1.89	30.53	0.31	1.02
**C18:1n9t**	*Trans*-oleic	2.18	0.04	1.91	0.20	0.04	19.96	0.08	BLOQ	-
**C18:1n9c**	*Cis*-oleic	4.36	0.09	1.97	8.24	0.19	2.30	9.05	0.15	1.65
**C18:2n6t**	*Trans*-linoleic	2.10	0.05	2.26	0.06	BLOQ	-	0.35	0.07	19.11
**C18:2n6c**	*Cis*-linoleic	2.19	0.04	1.96	22.79	0.24	1.04	38.07	0.46	1.20
**C18:3n6**	γ-Linoleic	2.11	0.04	2.13	0.06	BLOQ	-	ND	-	-
**C18:3n3**	α-Linoleic	2.17	0.05	2.18	0.25	0.09	37.65	1.68	0.24	14.24
**C20:0**	Arachidic	4.39	0.15	3.38	0.14	0.02	17.80	0.70	0.07	9.69
**C20:1**	Eicosenoic	2.11	0.05	2.26	0.19	0.04	19.57	0.38	0.04	11.53
**C20:2**	Eicosadienoic	2.12	0.05	2.38	0.39	0.02	4.63	0.25	0.02	9.82
**C20:3n6**	Dihomo-γ-linoleic	2.08	0.06	2.95	3.45	0.09	2.67	0.41	0.05	12.66
**C20:4n6**	Arachidonic	4.02	0.09	2.22	11.15	0.19	1.67	0.98	0.05	5.57
**C20:5n3**	Eicosapentaenoic	2.13	0.09	4.26	0.47	0.03	7.20	0.20	0.04	18.48
**C21:0**	Heneicosanoic	2.17	0.08	3.70	ND	-	-	0.01	BLOQ	-
**C22:0**	Behenic	4.32	0.18	4.19	0.26	0.03	13.02	0.09	BLOQ	-
**C22:1n9**	Erucic	2.09	0.06	2.73	0.02	BLOQ	-	0.01	BLOQ	-
**C22:2**	Brassic	2.09	0.09	4.13	ND	-	-	0.02	BLOQ	-
**C22:4**	Adrenic	1.77	0.06	3.60	0.51	0.02	4.60	ND	-	-
**C22:5n6**	Osbond	0.00	0.00	0.00	0.32	0.03	9.24	0.01	BLOQ	-
**C22:5n3**	Docosapentaenoic	1.88	0.08	4.20	0.89	0.06	6.53	0.21	0.03	15.68
**C22:6n3**	Docosahexaenoic	1.79	0.05	2.90	2.20	0.09	4.05	0.11	BLOQ	-
**C23:0**	Tricosanoic	2.15	0.09	4.35	0.11	BLOQ	-	0.03	BLOQ	-
**C24:0**	Lignoceric	4.30	0.20	4.57	0.27	0.03	10.47	0.13	BLOQ	-
**C24:1**	Nervonic	2.13	0.07	3.16	0.36	0.04	12.31	0.17	0.03	18.32

^a^Mixture of fatty-acid methyl ester standards; fresh batches of mixtures were prepared every 1 to 2 months, and mean values were calculated for each batch (see Methods section).

^b^Normal pooled human plasma (PLH-123-F; Sera Laboratories International).

^c^Normal pooled horse plasma (S-121-F; Sera Laboratories International).

^d^Mean level determined over 860 batches using three independent systems.

^e^Standard deviation determined over 860 batches using three independent systems.

^f^Coefficient of variation (100% × SD/mean).

^g^Not detected (level below 0.01%).

^h^Below limit of quantification (level below 0.15%). Mean values are given, but precision is insufficient.

### Throughput and speed

The aim of automating the method was not only to improve analytical precision, but more importantly to increase throughput and reliability, and to reduce the cost per sample. Analysis of fatty acids from the different fractions of human biological fluids is a laborious task. In previous projects, in which sample sets were analyzed using conventional manual methods, it was found that a team of two people could maintain a throughput of around 300 to 350 real samples per month. With the automated method running on three parallel systems, we achieved an average throughput of approximately 1,200 samples per month (including maintenance of the system, and reanalysis of failed samples). To run and maintain these three systems and cover all aspects from sample reception to manual checking of the chromatograms, we need a team of at least four people. This means that the actual throughput per person doubled by changing to the automated method, which significantly reduced the cost per sample. The systems proved to be adequately reliable in a research environment, with very limited downtime (less than 10%) and limited error rate (in almost all cases, the sample or extract could be recovered and useful results could be obtained after diagnosis of the reason for failure, modification of the method to accommodate the failure, and repeat analysis). However, the systems demanded constant preventive maintenance to maintain this level of reliability. As a result, the cost of spares for the sample-automation robotics was a significant contributor to the cost of ownership of this equipment. Nevertheless, this throughput allowed us to analyze 860 batches covering over 20,000 samples in 24 months.

## Conclusion

We have developed a convenient, high-throughput determination of phospholipid fatty acids, using a robot-based automated system combined with GC. Use of this method resulted in the reduction of the analytical burden and time. This method promises significant advantages for completing large sample numbers in major epidemiological studies.

Three parallel automated systems, which have proved to be both robust and user-friendly, are currently performing analysis of 90 samples plus standards and QC samples per day. The analytical method described here has sufficient reproducibility and long-term stability to be suitable for fatty-acid determination in plasma phospholipids for both epidemiological research studies and routine analysis. Furthermore, this method can easily be adapted to other samples, such as cell extracts, tissue homogenates, and food samples.

## Methods

### Equipment

Sequential multipurpose sampler (MPS) systems (Gerstel GmbH & Co. KG, Mülheim an der Ruhr, Germany) were used for sample preparation, subsequent phospholipid hydrolysis, FAME production, and injection of the sample into the GC for chromatographic analysis. The MPS systems were equipped with appropriately sized motorized syringes and accessories suitable for the tasks to be performed. The function of the MPS systems was controlled either by stand-alone Maestro software (Gerstel GmbH & Co. KG) for sample extraction and solid-phase cleanup, or Maestro software integrated within Agilent ChemStation software (Agilent Technologies, Inc., Wilmington, DE, USA) for FAME preparation and subsequent analysis. GC analysis was performed using a gas chromatograph (7890N; Agilent Technologies), equipped with an FID and split/splitless injector system. The separation was performed on a capillary column 30 m in length, with internal diameter of 0.25 mm, and film thickness of 0.2 μm (J&W HP-88; Agilent Technologies). A multi-tube vortexer (IKA Werke Gmbh & Co KG, Staufen Germany) and a refrigerated centrifuge (MSE Ltd, London, UK) were used for the manual liquid-liquid extraction. A vacuum manifold was used to assist the manual SPE, and a vacuum evaporator (AES2010; Savant Instruments Inc., Holbrook, NY, USA) was used to evaporate the solvents for the conventional method.

### Chemicals, standards, and QC samples

We used analytical reagent-grade chemicals and solvents: sodium chloride, chloroform, *n*-hexane, and 14% boron trifluoride (BF_3_)/methanol solution (Sigma-Aldrich,, St Louis, MO, USA). Methanol and acetone were HPLC grade (Thermo Fisher Scientific Inc., Rockford, IL, USA). All FAME standards (for complete list of fatty acids analyzed with full IUPAC names, see Additional file 1, Table S2) were were commercial grade (Supelco, Bellefonte, PA, USA). Aminopropylsilica 100 mg SPE cartridges (BondElut; Agilent Technologies) were used for the conventional method and Na_2_SO_4 _50 mg/NH_2 _100 mg SPE cartridges (BE Gerstel; Agilent Technologies) were used for the automated method. The QC samples used in this study were QC1, which was normal human plasma (mixed gender, pooled; PLH-123-F; Sera Laboratories International Ltd, Haywards Heath, West Sussex, UK) and QC2, which was normal pooled horse plasma (S-121-F; Sera Laboratories International). The QC1 sample was chosen to represent the samples of the application study [17], while the QC2 sample was chosen to be significantly different from QC1.

### Preparation of standards and QC materials

A series of working standards containing 37 FAMEs were prepared by accurately transferring 0.01, 0.05, 0.1, 0.5, and 1.0 ml each of the following individual standards and mixture of standards to glass vials: 37 mix FAME standard (47885-U; Supelco), C22:5n3 FAME (0.6 mmol/l), C22:4 FAME (0.75 mmol/l) and C22:5n6 FAME (0.6 mmol/l). C4:0 and C6:0 FAMEs presented in the mixture of 37 FAME standards overlapped with the solvent peak, and C22:3n3 and C20:4n6 did not separate under the chromatographic conditions used, leaving 37 FAMEs for quantification. The contents of the vials were evaporated to dryness under a stream of nitrogen, and then reconstituted in n-hexane (1 ml) to yield a series of the 37 FAME mixtures as the standards for GC calibration (for the complete list of all 37 fatty acids analyzed, see Additional file 1, Table S2).

Sufficient volumes of each QC material were homogenized, and aliquots of QC materials were transferred into cryo-tubes and stored at -80°C before analysis. QC1 and QC2 samples were analyzed in each batch to monitor inter-assay variation.

### Sample preparation

The Folch extraction for total lipids [20] and the isolation of phospholipid fraction (based on Burdge *et al. *[21]), performed by both the conventional and automated methods, are described below.

### Conventional manual sample extraction and SPE method

Aliquots of thawed plasma and QC samples (125 or 250 μl) were transferred to disposable glass tubes (16 × 100 mm). All sample volumes were made up to 0.5 ml with saline (0.9% w/v), then 1.0 ml methanol and 2.0 ml chloroform were added to extract total lipids from samples. The tubes were placed in a multi-tube vortex mixer, and mixed for 10 minutes at 1,200 rpm. Following this, 0.5 mlsodium chloride solution (1 mol/l) was added to the sample mixture, which was mixed by vortex for a further 2 minutes at 1200 rpm. Subsequent centrifugation at 2,500 rpm (1000 g) for 10 minutes was used to separate the organic and aqueous phases. The bottom chloroform layer was quantitatively transferred to another disposable glass tube (11 × 75 mm), then the tubes were placed in a vacuum evaporator for 45 minutes at 40°C to evaporate the solvent to near dryness.

Total lipid extract was dissolved in1.0 ml dry chloroform, and mixed by vortex. The sample was loaded on to a 1 ml aminopropyl SPE cartridge and allowed to slowly pass through the cartridge under gravity. Once the liquid meniscus reached the top of the SPE cartridge, the remaining liquid in the adsorbent bed was removed under vacuum. The SPE cartridge was then washed under vacuum with 2.0 ml chloroform. The phospholipid fraction was eluted into a 4 ml sample-collection vial with 2.0 ml of methanol chloroform (40:60 v/v) under vacuum, and the eluate was evaporated to dryness in a vacuum evaporator at 40 C. The 4 ml vials were capped and stored at -20°C until required for derivatization and analysis.

### Automated sample extraction and SPE method

The automated sample extraction and SPE method was constructed to replicate the existing manual method as far as possible, within the limitations of the robotic system,. The main limitation of the automated system was the ability to control only a single syringe, which necessitated extra syringe wash steps and the use of acetone as a co-solvent when changing between immiscible solvents, typically chloroform and water. In addition, vials of sizes appropriate for the hardware and the liquid volumes used at each stage were substituted for the disposable glass tubes used in the manual method. The plasma, saline, and methanol/chloroform mixtures were prepared off-line in 6 ml crimp-cap sealed vials. and the intermediate and finished products in 4 ml crimp-capped sealed vials (Chromacol; Esslab UK, Hadleigh Essex, UK).

**Figure 3:**
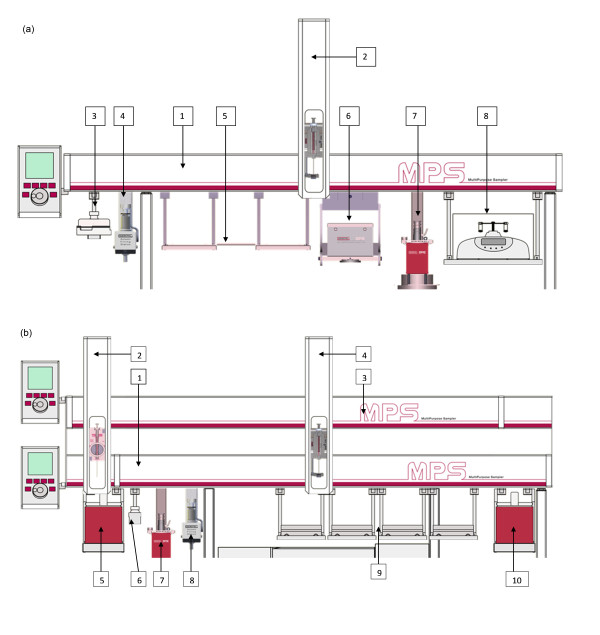
**Multipurpose sampler (MPS) systems for automated sample preparation and derivatization of fatty acids of the phospholipid fraction from human plasma samples**. **(a) **MPS single beam for phospholipid extraction. 1) Solid-phase extraction (SPE) MPS Beam; 2) syringe holder; 3) 1 mol/l saline reservoir; 4) solvent reservoirs; 5) three-position tray holder, 6) SPE cartridge tray; 7) SPE/blowdown; 8) vortex/centrifuge. **(b)**) Dual-beam MPS system for phospholipid hydrolysis, derivatization and injection. 1) Derivatization MPS beam; 2) derivatization syringe holder; 3) injection MPS beam; 4) injection syringe holder; 5) heated zone; 6) wash vials; 7) SPE/blowdown; 8) solvent reservoirs; 9) four-position tray holder; 10) agitator.

Set out below is a brief, step-by-step representation of the automated SPE procedure using the MPS system (numbers in brackets correspond to those in Figure 3a).

1. Pipette 250 μl thawed plasma into a vial. Add 250 μl 0.9% saline, 1.0 ml methanol, and 2.0 ml chloroform, then cap the vial. Repeat for 28 samples.

2. Pipette 500 μl 0.9% saline into a vial. Add 1.0 ml methanol and 2.0 ml chloroform, then cap the vial. This vial forms the process blank.

3. Pipette 250 μl thawed pooled human plasma into a vial. Add 250 μl 0.9% saline, 1.0 ml methanol, and 2.0 ml chloroform, then cap the vial. This vial is the QC1 sample.

4. Pipette 250 μl thawed horse plasma into a vial. Add 250 μl 0.9% saline, 1.0 ml methanol, and 2.0 ml chloroform, then cap the vial. This vial is the QC2 sample.

5. Shake all vials for 10 minutes in an orbital shaker [8].

6. Add 500 μl saline 1 mol/l by [2] from [3].

7. Shake for 30 seconds on [8].

8. Centrifuge at 2,700 rpm (500 g) for 5 minutes to promote phase separation on [8].

9. Aspirate 1,600 μl of the lower chloroform layer, and transfer to a clean vial by [2] from [4].

10. Dry the crude extract under a stream of nitrogen at 55°C on [7].

11. Reconstitute the crude extract in 2.0 ml dry chloroform by [2] from [4].

12. Pass the crude extract through a 1 ml aminopropyl silica cartridge on [7].

13. Wash the cartridge with 2.0 ml dry chloroform by [2] from [4].

14. Remove excess solvent from the sorbent bed by flushing with 4 × 2.5 ml aliquots of air on [7].

15. Elute the plasma-phospholipid fraction using 2.0 ml methanol/chloroform 40% v/v by [2] from [4].

16. Recover remaining elution solvent from the sorbent bed by flushing with 4 × 2.5 ml aliquots of air by [2] from [4].

17. Blow the phospholipid fraction dry under a stream of nitrogen at 55°C on [7].

18. Store the vials containing the phospholipid fraction at -20°C until required.

A schematic diagram of the timeline of the SPE analysis is shown (see Additional file 1, Figure S4).

### Automated preparation of FAME derivatives and GC analysis

A dual-rail preparation station (Gerstel MPS Prepstation; Gerstel GmbH & Co. KG) was located on a GC (7890N; Agilent Technologies). The lower rail was configured to perform the on-line phospholipid hydrolysis and FAME-derivative preparation, and the upper rail was configured to perform sample injection. For each experiment, 32 vials (4 ml each; comprising 29 dried phospholipid extracts, process blank, QC1 & QC2) were manually loaded on the Gerstel automation platform. Derivatization reagent (500 μl of 14% BF_3_/methanol solution), which both hydrolyses the phospholipids and forms the methyl esters of the liberated fatty acids, was added to the vials. The vials were incubated at 75°C for 45 minutes to yield the crude FAME derivatives. After the addition of 1 ml hexane to extract the FAMEs and 1 ml water to decompose any unreacted boron trifluoride and to scavenge any polar impurities, the vial was shaken to complete the FAME extraction. A portion (600 μL) of the upper hexane layer was transferred to a 1.5 ml high-recovery GC vial and evaporated to dryness. FAMEs were reconstituted in hexane 100 μL as the final sample solution. A 1 μL aliquot of this sample solutionwas injected into the GC split injector (split ratio 20:1) for GC analysis, using the following conditions: helium carrier 1.5 ml/min, oven temperature profile initially 120°C for 1 minute, then ramped to 170°C at 10°C/min, held for 6 minutes, and finally raised to 210°C at a rate of 3°C/min and held for 1 minute. The injector temperature was maintained at 250°C and the detector temperature maintained at 300°C. A post-run period (235°C for 3.5 minutes at 2.5 ml/min) was used to eliminate possible interferences. The total run time was approximately 30 minutes.

Separation of the 37 FAME standards was carried out using a GC capillary column (HP-88; Agilent Technologies). In the chromatogram, all compounds in the standard mixture were well resolved (see Additional file 1, Figure S1) with the exception of C20:3n3 and C20:4n6, which co-eluted, however, the level of C20:3n3 in human samples is negligible, and therefore we annotated this peak as C20:4n6 [22]. A representative plasma chromatogram separated on the HP88 column (see Additional file 1, Figure S2) showed that the *cis*/*trans *isomers and highly unsaturated fatty-acid mixtures were fully separated from the sample. A process blank chromatogram (see Additional file 1, Figure S3) was used to monitor any fatty acids contamination from chemicals, glassware, and the sample-preparation process. The process blank values (mainly trace levels of C14:0, C16:0, and C18:0) were deducted from the calculation of the plasma phospholipid fatty-acid results. Data collection and integration were performed with Agilent ChemStation software on a desktop computer.

Set out below is a brief, step-by-step representation of sample hydrolysis and derivatization by the automated method using the MPS dual-beam system (numbers in brackets correspond to Figure 1b).

1. Add 500 μl 14% BF_3_/MeOH solution to a vial containing the dried phospholipid fraction by [2] from [8].

2. Transfer vial to heated zone at 75°C and allow to react for 45 minutes [5].

3. Remove vial from heated zone and allow to cool to room temperature [9].

4. Add 1 ml hexane and 1 ml water by [2] from [8].

5. Shake for 5 minutes [10].

6. Stand for 2 minutes to allow phases to separate [9].

7. Remove 600 μl of the hexane layer containing the FAMEs, and transfer to a vial from which the injection is to be made by [2] from [8].

8. Dry the FAME extract under a stream of nitrogen at 35°C [7].

9. Reconstitute the extract in 100 μl hexane by [2] from [8].

10. Inject sample into GC by [4].

A schematic diagram of the timeline of sample hydrolysis and derivatization is shown (see Additional file 1, Figure S5).

### Identification and quantifiation

FAMEs in the samples were identified by comparison of their retention times with those of individual FAME standards. The palmitic acid methyl ester (C16:0) was used as a reference FAME. We measured the relative quantities of the 37 fatty acids, with each of the 37 analytes being measured as a percentage of the total fatty-acid signal.

### Statistical analysis

Linear regression, mean, and SD were calculated(Excel™ 2010; Microsoft Corp., Redmond, WA, USA). The precision of the assay was defined as the CV of at least six repeats of the QC sample across as many batches. For direct comparison, the Student's *t*-test was used. Single-factor ANOVA) was used for comparing more than two mean values.

## List of abbreviations

Avg: average line; CV: coefficient of variation; DHA: docosahexaenoic (C(22,6n3)); EPA: eicosapentaenoic (C(20,5n3)); FAME: Fatty-acid methyl ester; FID: Flame ionization detector; GC: Gas chromatography; IQC: Internal Quality Control; LCL: Lower control limit; LOD: Limit of detection; LOQ: Limit of quantification; MPS: Multipurpose samplers; QC1: Quality control; SPE: Solid-phase extraction; UCL: Upper control limit.

## Competing interests

The authors declare that they have no competing interests.

## Authors' contributions

LW, KS, and AK developed the study methodology; LW, KS, CC, IM, SY, and AK were responsible for data acquisition; AK, PP, ME, SY, and NF conceived of the study and participated in its design; SY, NF, and AK coordinated the study; LW drafted the manuscript; and KS, NF, and AK revised the manuscript. All authors read and approved the final manuscript for publication.

## Supplementary Material

Additional File 1 **Supplementary tables and figures**: Table S1: Fatty-acid profiles (%) of phospholipids in a quality control (QC2) sample, comparing conventional manual versus automated sample-preparation methods. Table S2: Instrumental validation using a fatty-acid methyl ester (FAME) standard mixture. Table S3: Recovery evaluation of human plasmas spiked with tknown phospholipids (C15:0 and *cis*-C18:2n6). Figure S1: A mixture standard of FAMEs separated on the HP88 column. Figure S2: A representative plasma chromatogram separated on the HP88 column. Figure S3: A reagent blank chromatogram separated on the HP88 column. Figure S4: SPE Timeline graphics. Figure S5: Derivatization and gas chromatography (GC) run timeline. Click here for file

## References

[B1] Colditz G Manson J Stampfer M Rosner B Willett W Speizer F Diet and risk of clinical diabetes in women. Am J Clin Nutr 1992 55 5 1018 1023 1315120 10.1093/ajcn/55.5.1018

[B2] Hu FB van Dam RM Liu S Diet and risk of type II diabetes: the role of types of fat and carbohydrate. Diabetologia 2001 44 7 805 817 10.1007/s001250100547 11508264

[B3] van Dam RM Willett WC Rimm EB Stampfer MJ Hu FB Dietary fat and meat intake in relation to risk of type 2 diabetes in men. Diabetes Care 2002 25 3 417 424 10.2337/diacare.25.3.417 11874924

[B4] Salmerón J Hu FB Manson JE Stampfer MJ Colditz GA Rimm EB Willett WC Dietary fat intake and risk of type 2 diabetes in women. Am J Clin Nutr 2001 73 6 1019 1026 11382654 10.1093/ajcn/73.6.1019

[B5] Hodson L Skeaff CM Fielding BA Fatty acid composition of adipose tissue and blood in humans and its use as a biomarker of dietary intake. Prog Lipid Res 2008 47 5 348 380 10.1016/j.plipres.2008.03.003 18435934

[B6] Patel PS Sharp SJ Jansen E Luben RN Khaw K-T Wareham NJ Forouhi NG Fatty acids measured in plasma and erythrocyte-membrane phospholipids and derived by food-frequency questionnaire and the risk of new-onset type 2 diabetes: a pilot study in the European Prospective Investigation into Cancer and Nutrition (EPIC)-Norfolk cohort. Am J Clin Nutr 2010 92 5 1214 1222 10.3945/ajcn.2010.29182 20861175

[B7] Mozaffarian D Cao H King IB Lemaitre RN Song X Siscovick DS Hotamisligil GS Circulating palmitoleic acid and risk of metabolic abnormalities and new-onset diabetes. Am J Clin Nutr 2010 92 6 1350 1358 10.3945/ajcn.110.003970 20943795 PMC2980960

[B8] Illig T Gieger C Zhai G Römisch-Margl W Wang-Sattler R Prehn C Altmaier E Kastenmüller G Kato B Mewes H-W Meitinger T de Angelis M Kronenberg F Soranzo N Wichmann HE Spector T Adamski J Suhre K A genome-wide perspective of genetic variation in human metabolism. Nat Genet 2010 42 137 178 a7312bb2-13a4-8015-6a0a-d2b371048555 10.1038/ng.507 20037589 PMC3773904

[B9] Suhre K Shin S-Y Petersen A-K Mohney R Meredith D Wägele B Altmaier E Gram Cardio Deloukas P Erdmann J Grundberg E Hammond C de Angelis M Kastenmüller G Köttgen A Kronenberg F Mangino M Meisinger C Meitinger T Mewes H-W Milburn M Prehn C Raffler J Ried J Römisch-Margl W Samani N Small K Wichmann HE Zhai G Illig T Human metabolic individuality in biomedical and pharmaceutical research. Nature 2011 477 7362 54 60 10.1038/nature10354 21886157 PMC3832838

[B10] Tanaka T Shen J Abecasis G Kisialiou A Ordovas J Guralnik J Singleton A Bandinelli S Cherubini A Arnett D Tsai M Ferrucci L Genome-wide association study of plasma polyunsaturated fatty acids in the InCHIANTI Study. PLoS Genet 2009 5 1 e1000338 10.1371/journal.pgen.1000338 19148276 PMC2613033

[B11] Welch AA Shakya-Shrestha S Lentjes MA Wareham NJ Khaw K-T Dietary intake and status of n-3 polyunsaturated fatty acids in a population of fish-eating and non-fish-eating meat-eaters, vegetarians, and vegans and the precursor-product ratio of α-linolenic acid to long-chain n-3 polyunsaturated fatty acids: results from the EPIC-Norfolk cohort. Am J Clin Nutr 2010 92 5 1040 1051 10.3945/ajcn.2010.29457 20861171

[B12] Masood M Salem N High-throughput analysis of plasma fatty acid methyl esters employing robotic transesterification and fast gas chromatography. Lipids 2008 43 2 171 180 10.1007/s11745-007-3130-9 18084789

[B13] Lin Y Salem N Wells E Zhou W Loewke J Brown J Lands W Goldman L Hibbeln J Automated high-throughput fatty acid analysis of umbilical cord serum and application to an epidemiological study. Lipids 2012 47 5 527 539 10.1007/s11745-012-3661-6 22430941 PMC3475606

[B14] Glaser C Demmelmair H Koletzko B High-throughput analysis of fatty acid composition of plasma glycerophospholipids. J Lipid Res 2010 51 1 216 221 10.1194/jlr.D000547 19654422 PMC2789782

[B15] Duong C Roper M A microfluidic device for the automated derivatization of free fatty acids to fatty acid methyl esters. Analyst 2012 137 4 840 846 10.1039/c2an15911b 22166918 PMC6746238

[B16] Klingler M Koletzko B Novel methodologies for assessing omega-3 fatty acid status - a systematic review. Br J Nutr 2012 107 Suppl 2 63 10.1017/S0007114512001468 22591903

[B17] The_InterAct_Consortium The InterAct Project: An examination of the interaction of genetic and lifestyle factors on the incidence of type 2 diabetes in the EPIC Study. Diabetologia 2011 54 9 11 10.1007/s00125-011-2182-9 PMC4222062 21717116

[B18] Dodds ED McCoy MR Rea LD Kennish JM Gas chromatographic quantification of fatty acid methyl esters: Flame ionization detection vs. electron impact mass spectrometry. Lipids 2005 40 4 419 428 10.1007/s11745-006-1399-8 16028722

[B19] Mohammed MA Cheng KK Rouse A Marshall T Bristol, Shipman, and clinical governance: Shewhart's forgotten lessons. Lancet 2001 357 9254 463 467 10.1016/S0140-6736(00)04019-8 11273083

[B20] Folch J Lees M Sloane Stanley GH A simple method for the isolation and purification of total lipides from animal tissues. J Biol Chem 1957 226 1 497 509 13428781

[B21] Burdge GC Wright P Jones AE Wootton SA A method for separation of phosphatidylcholine, triacylglycerol, non-esterified fatty acids and cholesterol esters from plasma by solid-phase extracti on. Br J Nutr 2000 84 5 781 787 11177194

[B22] Browning L Walker C Mander A West A Madden J Gambell J Young S Wang L Jebb S Calder P Incorporation of eicosapentaenoic and docosahexaenoic acids into lipid pools when given as supplements providing doses equivalent to typical intakes of oily fish. Am J Clin Nutr 2012 96 4 748 758 10.3945/ajcn.112.041343 22932281 PMC3441107

[B23] Van Batenburg M Coulier L van Eeuwijk F Smilde A Westerhuis J New figures of merit for comprehensive functional genomics data: the metabolomics case. Analytical Chemistry 2011 83 9 3267 3274 10.1021/ac102374c 21391558

