# Supplementary material for: Development and validation of a robust automated analysis of plasma phospholipid fatty acids for metabolic phenotyping of large epidemiological studies
Source: Genome Med. 2013 Apr 25;5(4):39. doi: 10.1186/gm443 (PMC3706814; doi:10.1186/gm443)
Supplement: Additional File 1 — Supplementary tables and figures: Table S1: Fatty-acid profiles (%) of phospholipids in a quality control (QC2) sample, comparing conventional manual versus automated sample-preparation methods. Table S2: Instrumental validation using a fatty-acid methyl ester (FAME) standard mixture. Table S3: Recovery evaluation of human plasmas spiked with tknown phospholipids (C15:0 and cis-C18:2n6). Figure S1: A mixture standard of FAMEs separated on the HP88 column. Figure S2: A representative plasma chromatogram separated on the HP88 column. Figure S3: A reagent blank chromatogram separated on the HP88 column. Figure S4: SPE Timeline graphics. Figure S5: Derivatization and gas chromatography (GC) run timeline. [file gm443-S1.PDF]

Development and validation of a robust automated analysis of plasma phospholipid fatty acids for metabolic phenotyping of large epidemiological studies

Laura Yun Wang[1], Keith Summerhill[1], Carmen Rodriguez-Canas[1], Ian Mather[1], Pinal Patel[2] Michael Eiden[1], Stephen Young[1], Nita G Forouhi[2] and Albert Koulman[1]*.*

[1] Medical Research Council Human Nutrition Research, Elsie Widdowson Laboratory, 120 Fulbourn Road, Cambridge, CB1 9NL, United Kingdom

[2] MRC Epidemiology Unit, Institute of Metabolic Science, Box 285, Addenbrooke's Hospital, Hills Road, Cambridge, CB2 0QQ United Kingdom

Table S1: Fatty acid profiles (%) of phospholipids in a quality control sample (QC2), comparing conventional manual method vs. automated sample preparation method.

	Conventional sample preparation ([a]n=26)			Automated sample preparation (n=26)		
Fatty acids	Mean	SD	CV (%)	Mean	SD	CV (%)
C15:0	0.15	0.01	8.74	0.14	0.01	9.19
C16:0	15.09	0.21	1.37	14.8	0.18	1.18
C16:1	0.34	0.01	4.02	0.33	0.01	3.04
C17:0	0.70	0.01	1.34	0.69	0.01	1.14
C18:0	29.97	0.23	0.75	29.78	0.22	0.74
C18:1n9t	9.01	0.11	1.21	9.03	0.08	0.90
C18:2n6t	0.38	0.01	3.38	0.37	0.01	3.12
C18:2n6c	38.06	0.27	0.72	38.30	0.19	0.49
C18:3n3	1.75	0.02	7.76	1.67	0.01	3.45
C20:0	0.73	0.13	2.16	0.74	0.06	1.86
C20:1	0.41	0.04	10.71	0.42	0.02	4.89
C20:2	0.24	0.01	2.60	0.25	0.01	2.43
C20:3n6	0.41	0.01	3.32	0.39	0.01	2.20
C20:4n6	0.98	0.02	2.16	0.97	0.03	2.79
C20:5n3	0.31	0.04	13.05	0.24	0.02	7.65
C22:5n3	0.26	0.03	11.92	0.20	0.01	5.29
C22:6n3	0.16	0.02	16.14	0.10	0.01	8.15
C24:0	0.13	0.02	17.33	0.12	0.01	5.40
C24:1	0.18	0.03	16.07	0.17	0.01	5.04

[a] n is the number of samples.

Supporting Information

Table S2: Instrumental validation using FAME standard mixture

Fatty acids	Trivial acid name[a]	IUPAC name[b]	Linearity ranges μmolL^{-1}, (r^2)[c]	Inter-precisions (n=56)[d] CV% Mix Std1	Mix Std2	Detection limits (ng) per inject	Limits of quantitation (μmolL^{-1})
C8:0	caprylic	octonoic	0 – 1387, (0.991)	4.7%	3.8%	0.3	1.4
C10:0	capric	decanoic	0 – 2322, (0.991)	2.8%	3.1%	0.3	1.5
C11:0	undecanoic	undecanoic	0 – 1074, (0.995)	2.8%	2.5%	0.5	2.2
C12:0	lauric	dodecanoic	0 – 1997, (0.997)	2.5%	2.2%	0.4	1.7
C13:0	tridecanoic	tridecanoic	0 – 933, (0.998)	2.4%	1.9%	0.6	2.3
C14:0	myristic	tetradecanoic	0 – 1751, (0.998)	2.0%	1.5%	0.5	1.8
C14:1	myristoleic	tetradecenoic	0 – 883, (0.998)	2.1%	1.9%	0.6	2.2
C15:0	pentadecanoic	pentadecanoic	0 – 825, (0.998)	1.7%	1.6%	0.6	2.1
C15:1	pentadecenoic	*cis*-10-pentadecenoic	0 – 832, (0.998)	1.9%	1.8%	0.6	2.1
C16:0	palmitic	hexadecanoic	0 – 2340, (0.999)	1.9%	2.6%	0.5	1.6
C16:1	palmitoleic	*cis*-9-hexadecenoic	0 – 786, (0.999)	1.8%	1.8%	0.6	2.0
C17:0	margaric	heptadecanoic	0 – 740, (0.999)	1.8%	2.5%	0.6	1.8
C17:1	heptadecenoic	*cis*-10-heptadecenoic	0 – 745, (0.999)	1.8%	1.8%	0.6	1.9
C18:0	stearic	octadecanoic	0 – 1406, (1.000)	2.7%	2.8%	1.4	4.1
C18:1n9t	*trans*-oleic	*trans*-9-octadecenoic	0 – 708, (1.000)	1.8%	1.7%	1.4	4.1
C18:1n9c	*cis*-oleic	*cis*-9-octadecenoic	0 – 1416, (1.000)	1.8%	1.8%	1.3	3.8
C18:2n6t	*trans*-linoleic	*trans*-9,12-octadecadienoic	0 – 713, (0.999)	1.9%	1.8%	1.4	4.2
C18:2n6c	*cis*-linoleic	*cis*-9,12-octadecadienoic	0 – 713, (1.000)	1.8%	1.8%	1.5	4.5
C18:3n6	γ-linoleic	*cis*-6,9,12-octadecatrieno	0 – 718, (1.000)	2.1%	2.3%	1.6	4.8
C18:3n3	α-linoleic	*cis*-9,12,15-octadecatrieno	0 – 718, (0.999)	3.1%	2.0%	1.3	3.9
C20:0	arachidic	eicosanoic	0 – 1280, (0.999)	4.2%	3.3%	1.3	3.5
C20:1	eicosenoic	*cis*-11-eicosenoic	0 – 950, (0.999)	1.9%	1.8%	1.4	5.5
C20:2	eicosadienoic	*cis*-11,14-eicosadienoic	0 – 648, (0.999)	1.9%	1.9%	1.1	3.0
C20:3n6	dihomo-γ-linoleic	*cis*-8,11,14-eicosatrienoic	0 – 653, (1.000)	2.3%	2.1%	1.5	4.1
C20:4n6	archidonic	*cis*-5,8,11,14-eicosatetraenoic	0 – 657, (0.999)	1.8%	2.0%	1.5	4.1
C20:5n3	eicosapentaenoic	*cis*-5,8,11,14,17-eicosapentaenoic	0 – 661, (0.999)	5.1%	3.9%	1.5	4.1
C21:0	heneicosanoic	heneicosanoic	0 – 613, (0.999)	5.1%	3.6%	1.1	2.8
C22:0	behenic	docosanoic	0 – 1175, (1.000)	5.3%	3.9%	1.4	3.4
C22:1n9	erucic	*cis*-13-docosenoic	0 – 591, (0.999)	2.2%	2.3%	1.5	3.7
C22:2	brassic	*cis*-13,16-docosadienoic	0 – 594, (1.000)	6.0%	3.9%	1.1	2.7
C22:4	adrenic	*cis*-4,10,13,16-Docosatetraenoic	0 – 752, (1.000)	2.6%	2.6%	1.8	4.5
C22:5n3	docosapentaenoic	*cis*-7,10,13,16,19-docopentaenoic	0 – 605, (0.999)	2.4%	2.5%	1.4	3.5

Fatty acids	Trivial acid name[a]	IUPAC name[b]	Linearity ranges $\mu molL^{-1}$, $(r^2)^c$	Inter-precisions (n=56)[d] CV%		Detection limits (ng) per inject	Limits of quantitation ($\mu molL^{-1}$)
				Mix Std1	Mix Std2		
C22:5n6	Osbond	*cis*-4,7,10,13,16-docopentaenoic	0 – 756, (0.999)	3.6%	3.9%	1.5	3.6
C22:6n3	docosahexaenoic	*cis*-4,7,10,13,16,19-docohexaenoic	0 – 609, (0.999)	3.1%	2.5%	1.4	3.6
C23:0	tricosanoic	tricosanoic	0 – 564, (0.999)	5.3%	4.1%	1.4	3.3
C24:0	lignoceric	tetracosanoic	0 – 1085, (1.000)	5.6%	4.2%	1.0	2.3
C24:1	nervonic	*cis*-15-tetracosenoic	0 – 546, (1.000)	2.5%	2.6%	1.0	2.3

[a] When there is no common trivial name the systematic name is used.

[b] International union of pure and applied chemistry (IUPAC).

[c] r^2 is the R-square of least squares regression line from the standard calibration curve.

[d] n is the number of batches.

Table S3: Recovery evaluation for the human plasmas spiked with the known phospholipids (C15:0 & cis C18:2n6)

Spiked plasma replicates	C15:0 recoveries (%)	Cis- C18:2n6 recoveries (%)
1	86.2	97.2
2	82.0	96.4
3	80.4	83.3
4	77.1	88.8
5	89.3	77.1
6	79.3	100.8
Mean	82.4	90.6
SD	4.5	9.2
CV%	5.5	10.1

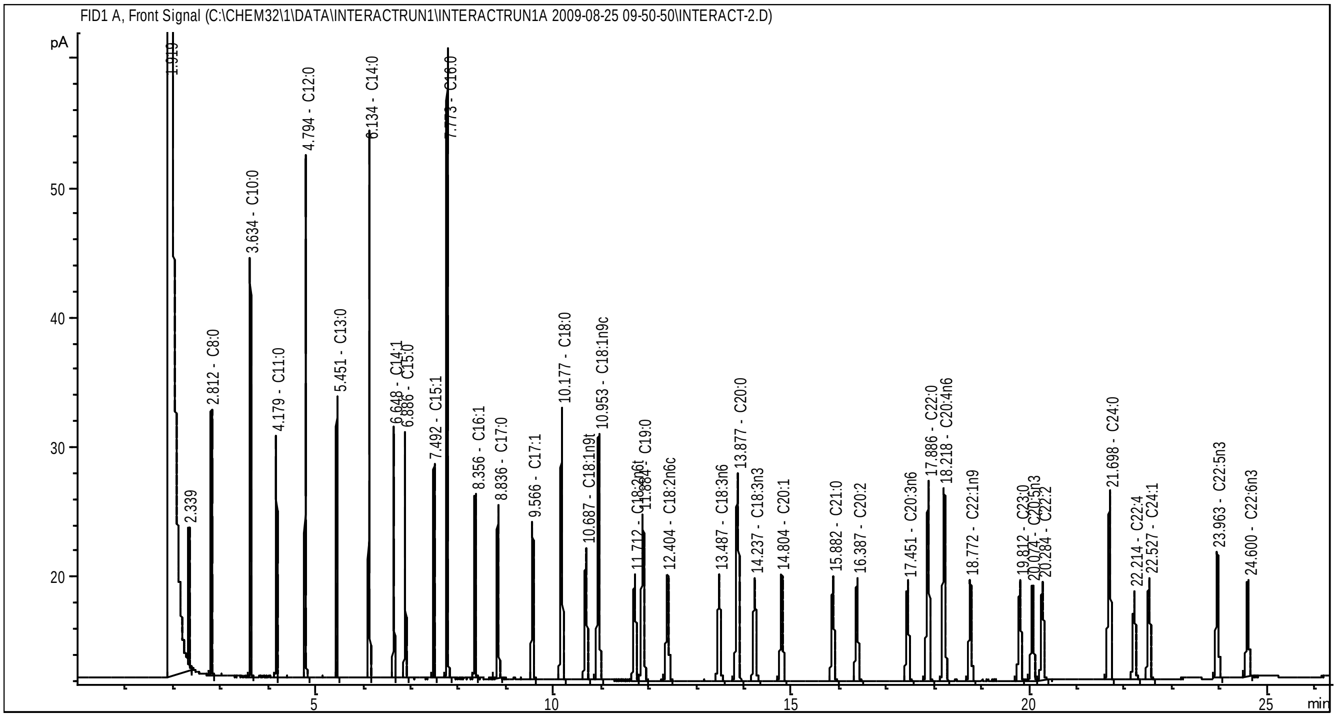

Figure S1: A mixture standard of fatty acid methyl esters separated on the HP88 column.

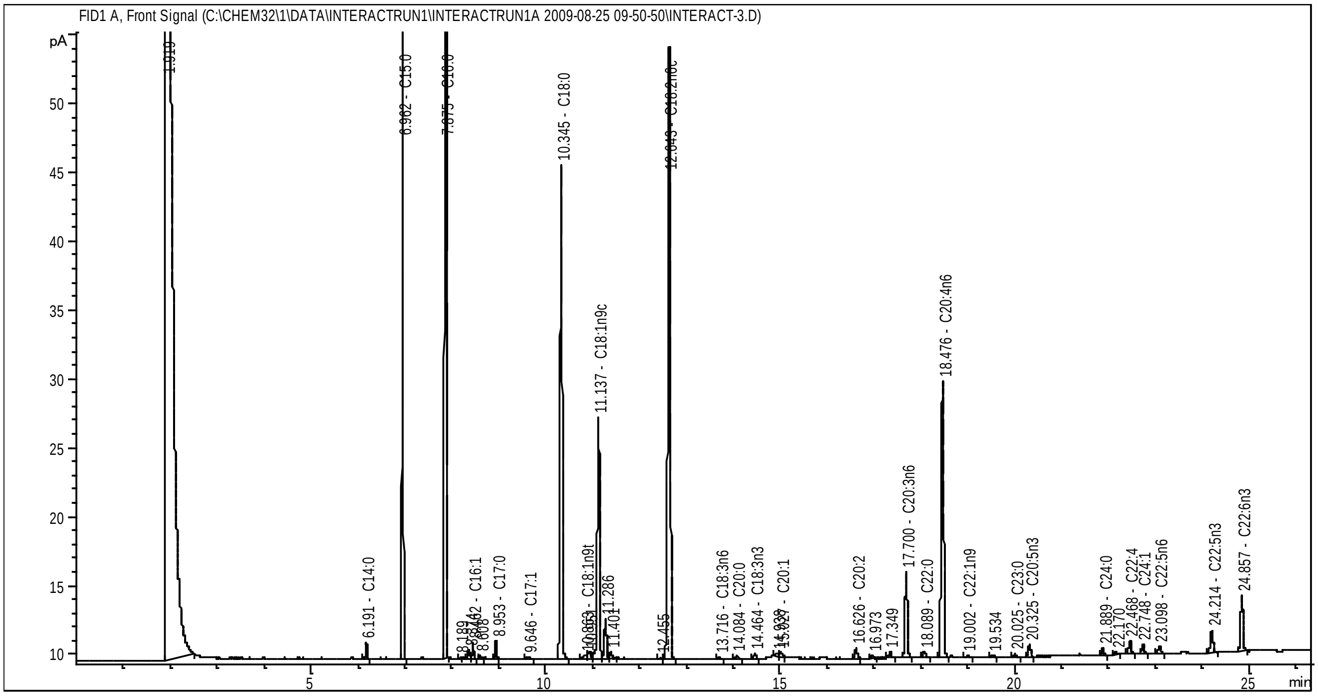

Figure S2: A representative plasma chromatogram separated on the HP88 column.

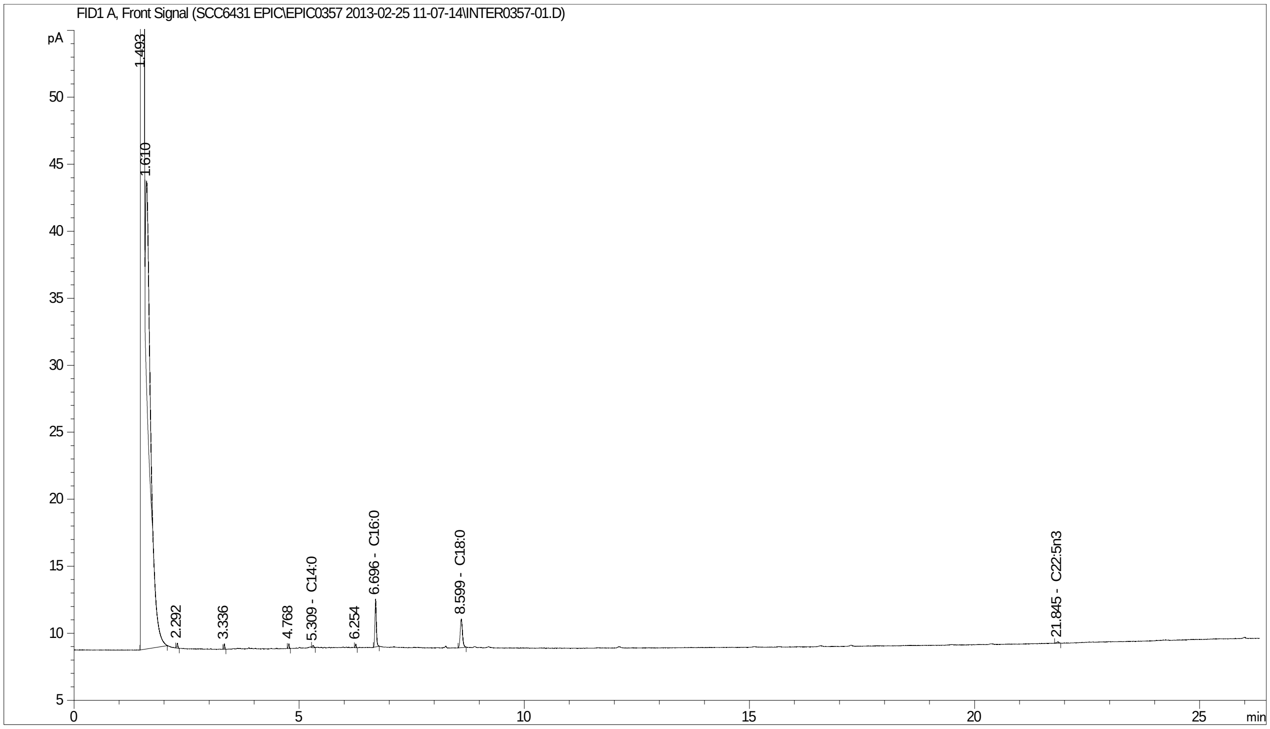

Figure S3: A reagent blank chromatogram separated on the HP88 column.

Figure S4: SPE Timeline Graphics

The Maestro software controlling the robots is able to display a predicted timeline from which the time to run a batch of samples can be estimated. In addition, the effect of changes to steps in the method can be assessed.

The coloured bands in the graphics shown below broadly relate to individual steps within the preparation method and the colour of the band gives an indication of the process undertaken within that band. The following key illustrates the colour/process correspondence for some of the steps:

Colour	Robot	Process
Green	Both	Addition or aspiration of solvent. May involve syringe washes.
Yellow	Both	Wait for specified time.
Pink	Both	Sample Evaporation
Orange	GC	Mix for specified time
Violet	GC	Injection syringe wash
Beige	GC	GC Run time plus GC Cool down time

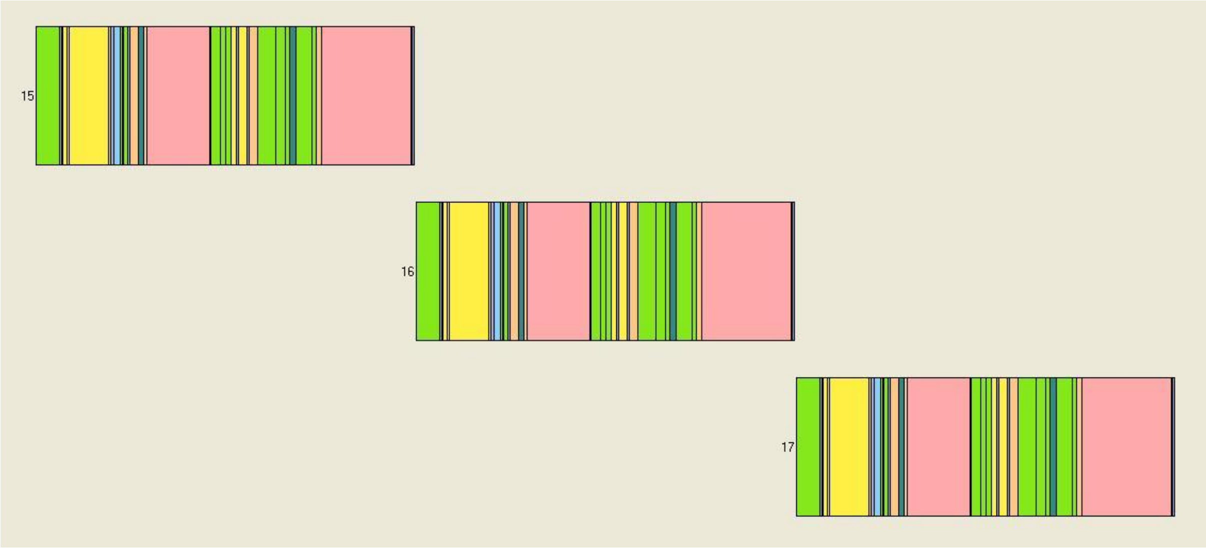

SPE Timeline.

Note that the SPE timeline obtained illustrates the processing of samples in a linear manner. One sample is processed from start to finish before the processing of the subsequent sample is commenced. As a guide, the time to completely process a single sample is approximately 45 minutes. Although the steps have not been explicitly described, the SPE sample cleanup occurs in the multiple narrow bands between the pink sample evaporation steps.

Figure S5: Derivatisation & GC Run Timeline

The Maestro software controlling the robots is able to display a predicted timeline from which the time to run a batch of samples can be estimated. In addition, the effect of changes to steps in the method can be assessed.

The coloured bands in the graphics shown below broadly relate to individual steps within the preparation method and the colour of the band gives an indication of the process undertaken within that band. The following key illustrates the colour/process correspondence for some of the steps:

Colour	Robot	Process
Green	Both	Addition or aspiration of solvent. May involve syringe washes.
Yellow	Both	Wait for specified time.
Pink	Both	Sample Evaporation
Orange	GC	Mix for specified time
Violet	GC	Injection syringe wash
Beige	GC	GC Run time plus GC Cool down time

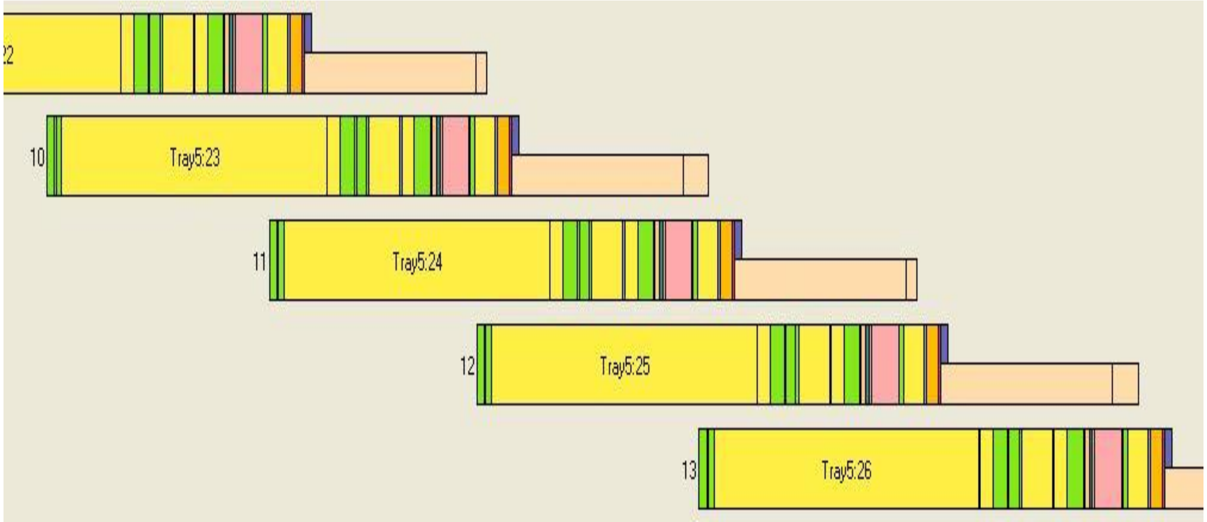

Note that the Derivatisation & GC Run timeline is able to process multiple samples simultaneously as shown by the overlap in the individual timelines in the above graphic. The protocol in use allows a maximum of four samples to be in process simultaneously. The interleaving obtained is a result of considerable method optimisation to ensure that there is sufficient time available in the processing of one sample to allow a process in a subsequent sample to be 'fitted in' to that time. This required the possibly counter-intuitive increase of certain process step times to obtain more efficient interleaving which, in turn, led to a net reduction in the time to process a sample batch.

The target was to have the GC run plus cool down time as the overall rate determining step for the process and this objective is almost achieved in that, while the processing of an individual sample takes in the region of 90 minutes, a GC injection is performed about twice per hour.
